# The impact of SARS-CoV-2 (COVID-19) pandemic on trauma bay management and guideline adherence in a European level-one-trauma centre

**DOI:** 10.1007/s00264-020-04740-5

**Published:** 2020-07-28

**Authors:** Sascha Halvachizadeh, Michel Teuben, Till Berk, Valentin Neuhaus, Hans-Christoph Pape, Roman Pfeifer

**Affiliations:** 1 Department of Trauma, University Hospital Zurich, Rämistrasse 100, 8091 Zurich, Switzerland; 2 Harald-Tscherne laboratory for orthopedic and trauma research, University of Zurich, Sternwartstrasse 14, 8091 Zurich, Switzerland

**Keywords:** Trauma bay management, Severe trauma, COVID-19, SARS CoV-2, Polytrauma management

## Abstract

**Purpose:**

SARS CoV-2 (COVID-19) represents a pandemic that has led to adjustments of routine clinical practices. The initial management in the trauma bay follows detailed international valid algorithms. This study aims to work out potential adjustments of trauma bay algorithms during a global pandemic in order to reduce contamination and to increase safety for patients and medical personnel.

**Methods:**

This retrospective cohort study compared patients admitted to the trauma bay of one academic level-one trauma centre in March and April 2019 with patients admitted in March and April 2020. Based on these datasets, possible adjustments of the current international guidelines of trauma bay management were discussed.

**Results:**

*Group Pan* (2020, *n* = 30) included two-thirds the number of patients compared with *Group Ref* (2019, *n* = 44). The number of severely injured patients comparable amongst these groups: mean injury severity score (ISS) was significantly lower in Group Pan (10.5 ± 4.4 points) compared with Group Ref (15.3 ± 9.2 points, *p* = 0.035). Duration from admission to whole-body CT was significantly higher in Group Pan (23.8 ± 9.4 min) compared with Group Ref (17.3 ± 10.7 min, *p* = 0.046). Number of trauma bay admissions decreased, as did the injury severity for patients admitted in March and April 2020. In order to contain spreading of SARS Cov-2, the suggested recommendations of adjusting trauma bay protocols for severely injured patients include (1) minimizing trauma bay team members with direct contact to the patient; (2) reducing repeated examination as much as possible, with rationalized use of protective equipment; and (3) preventing potential secondary inflammatory insults.

**Conclusion:**

Appropriate adjustments of trauma bay protocols during pandemics should improve safety for both patients and medical personnel while guaranteeing the optimal treatment quality. The above-mentioned proposals have the potential to improve safety during trauma bay management in a time of a global pandemic.

## Background

Trauma is amongst the top ten causes of death worldwide [1]. Numerous international guidelines have been introduced worldwide in order to standardize the treatment of severely injured patients and to improve their survival [2–4]. These guidelines include recommendations for the minimum number of required medical professionals in the trauma bay (e.g., anesthesiologists, traumatologist, neurosurgeons, nurses, technicians etc..), initial assessments according to Advanced Trauma Life Support (e.g., blood pressure measurements, respiratory rate, auscultation and examination of body regions, etc.), and settings around the trauma bay management, such as operation room or computer tomography availability [5, 6]. SARS CoV-2, a rapidly spreading pandemic, has led to increasing infection and mortality rates which threatens to overwhelm the critical care infrastructure [7]. Several measures have recently been implemented in order to optimize treatment and outcomes of COVID-19 patients. Both intramural (mandated quarantine of medical personnel, novel isolation wards, involvement of other physicians from other specialties in daily COVID-19 care) and extramural (businesses have been closed, social distancing, isolation) measures have been introduced. Reports documenting infections have shown symptoms or even mortalities of medical personnel (nurses and doctors) and healthcare workers exacerbating the crisis [8]. Additional stresses include the increased need for medical protective equipment and medical supplies. Without a doubt, the increased spread of the COVID-19 over the population and lower capacities in critical care infrastructure directly affects the management and strategies in treatment of severely injured patients.

This study aims to analyze the impact of the global SARS CoV-2 pandemic on the treatment of patients in the trauma bay in a level-one trauma center. Based on the challenges brought by SARS CoV-2, this study further discusses potential adjustments of trauma bay guidelines:

1. How to comply with international recommendations and guidelines and reduce risks of infections in medical personal (surgeons, nurses, emergency practitioners) involved in trauma management.
2. How to maintain the quality of trauma management without being contaminated or spreading the virus throughout the hospital.
3. How to protect of critical care infrastructure from becoming overwhelmed.

## Methods

The local institutional review board and the cantonal ethics committee received a detailed study protocol and waved the requirement for ethical approval to conduct this study (Nr. 2020-00789). This retrospective cohort study was conducted under adherence to the STROBE guidelines [9].

### Study population and data collection

Data from patients who were treated in the trauma bay of one academic level-one trauma center was extracted from the electronic patient charts. For the purpose of the study, two study groups were composed based on admission data:

- *Group Pan(demic)*: included all patients admitted during the COVID-19 pandemic period between Mar 01, 2020 and Apr 15, 2020.
- *Group Ref(erence)* was composed to serve as a reference group and included all patients admitted between Mar 01, 2019 and Apr 15, 2019.

Exclusion criteria were death in the trauma bay, secondary admissions to the trauma from other hospitals, or international repatriations. This study compared patients treated in the same month to increase seasonal comparability and to reduce selection bias. The patients included were treated for the following reason: The Swiss Federal Department of Health recommended in March 2020 behavioral changes of the population to reduce the spreading of SARS CoV-2. These recommendations included social distancing of 2 m (6 ft), staying at home as much as possible, and short-time contact to other persons (15 min). In addition, non-essential businesses have been closed. For clinical personnel, wearing of personal protective equipment, such as gloves, masks, coats, and glasses has been introduced.

### Definitions

The Injury Severity Score (ISS) was calculated based on information found on the discharge paper in common manner [10]. Severely injured patients were defined as ISS ≥ 16 points [2]. Total duration in the trauma bay indicates the time from admission to the trauma bay to the time of discharge to either the operating room (OR), intensive care unit (ICU), or the ward. The time from admission to whole-body CT scan (WBCT) represents the delivery procedure, an initial assessment, and the preparation of the trauma team and the patient for WBCT. Life-saving interventions included emergency intubation, placement of a chest tube, laparotomy, craniotomy, thoracotomy, external fixation of pelvic fracture, and other interventions to stop major bleedings in the trauma bay. Injury mechanisms included fall from any height, motor vehicle accidents (MVAs), blunt trauma from other causes (violent attacks), penetrating injuries, or suicide attempts. Treatment and diagnostics as well as trauma team members followed the local and international guidelines for trauma resuscitation [6].

### Parameters studied

Collected variables included patient demographics (age, gender), ISS, total duration spent in the trauma bay, duration from admission WBCT, number of severely injured patients, and life-saving interventions performed in the trauma bay.

### Statistical analysis

Continuous variables were summarized as mean ± standard deviation and discrete variables as a number and percentage. Group comparison was performed using Student’s *t* test on continuous and Pearson’s chi-square test on discrete variable with 95% confidence interval (CI) in case of normal distribution. Skewed distributed variables were compared using Mann-Whitney *U* test. A *p* value of below 0.05 was considered statistically significant. All statistical analyses were performed using R (R Core Team (2018). R: A language and environment for statistical computing. R Foundation for Statistical Computing, Vienna, Austria. URL https://www.R-project.org/).

## Results

### Patient characteristics

A total of 76 patients were initially identified. One patient was excluded in each study group, due to death before finishing trauma bay diagnostics. In *Group Ref*, one patient died after a self-inflicted gunshot to the head, and in *Group Pan*, one patient died due to brain herniation after suicidal hanging.

The final cohort included 74 patients, mean age 50.0 years (± 21.8), with a majority being male (*n* = 54, 73.0%) (Table 1). *Group Ref* included 44 (59.4%) patients, while *Group Pan* included 30 (40.5%) patients. Demographic variables including age and gender distribution were comparable amongst these groups.

**Table 1:** Included patients

Number	74
Age (years), mean (SD)	50.0 (21.8)
Male, *n* (%)	54 (73.0)
Patients during COVID-19 Pandemic, *n* (%)	30 (40.5)
ISS (points), mean (SD)	12.2 (8.3)
Severely injured patients, *n* (%)	22 (29.7)
Total duration in trauma bay (min), mean (SD)	83.3 (30.4)
Duration from admission to WBCT (min), mean (SD)	19.1 (10.3)

*SD* standard deviation, *ISS* Injury Severity Score, *WBCT* whole-body CT

### Injury severity and mechanism

Falls were the most common injury mechanism (*Group Ref n* = 23 (52.3%) vs. *Group Pan n* = 17 (56.7%), 0.912), followed by motor vehicle accidents (*Group Ref n* = 16 (36.4%) vs. *Group Pan n* = 11 (36.7%), 0.912). The number of severely injured patients in both groups was comparable (*Group Ref n* = 15 (34.1%) versus *Group Pa*n = 7 (23.3%), 0.462). However, the mean ISS was significantly higher in *Group Ref* (15.3 ± 9.2 points) compared with Group Pan (10.5 ± 64.4 points, 95% CI 0.8 to 6.4, *p* = 0.035) (Table 2).

**Table 2:** Comparison of time spent in trauma bay

	Group Ref	Group Pan	*p* value
*n*	44	30	
Age (years), mean (SD)	50.7 (23.1)	48.9 (20.1)	0.739
Male, *n* (%)	32 (72.7)	22 (73.3)	1
ISS (points), mean (SD)	15.3 (9.2)	10.5 (4.4)	0.035
Severely injured patients, *n* (%)	15 (34.1)	7 (23.3)	0.462
Total duration in trauma bay (min), mean (SD)	76.2 (29.6)	93.6 (28.9)	0.014
Duration from admission to WBCT (min), mean (SD)	17.3 (10.7)	23.8 (9.4)	0.046

*n* number, *SD* standard deviation, *ISS* Injury Severity Score, *WBCT* whole-body CT

### Trauma bay times

The total duration in the trauma bay for *Group Ref* was 76.2 ± 29.6 minutes while in *Group Pan*, the total duration was 93.6 ± 28.9 min. This difference was statistically significant (95% CI − 31.2 to − 3.5, 0.014).

The duration from admission to WBCT for *Group Ref* was 17.3 ± 10.7 minutes. This was found to be significantly lower when compared with *Group Pan* (23.8 ± 9.4 minutes, 95% CI − 9.2 to − 1.2, *p* = 0.046).

### Lifesaving interventions

The total number of life-saving interventions per patient in the trauma bay was comparable amongst *Group Ref* and *Group Pan* (0.6 ± 0.9 vs. 0.4 ± 0.8, 95%CI -0.2 to 0.6, 0.31). After treatment in the trauma bay, the patients were frequently dismissed to the ICU (*Group Ref n* = 25 (56.8%) vs *Group Pan n* = 18 (60%), 0.974).

## Discussion

The SARS CoV-2 pandemic challenges routine clinical care and more specifically largely interferes with trauma care. In order to optimize planning of trauma care in the near future, there is a need to define specific alterations and to identify practical pitfalls. To do so, the current study aimed to compare trauma bay characteristics under COVID-19 pandemic conditions and during a corresponding time period under regular circumstances (prior to COVID-19 pandemic). The following differences were identified:

- During the pandemic, there were 31.2% less patients treated in the trauma bay
- Despite a comparable number of severely injured patients, the Injury Severity Score was significantly lower during the pandemic
- Both the total duration in the trauma bay and the duration from admission to WBCT were significantly higher during the pandemic

The observed decrease in trauma bay admissions during the COVID-19 pandemic is likely associated with the restrictions introduced by the federal health department. Individuals were recommended to avoid unnecessary movements and expected to stay at home, as much as possible. Furthermore, non-essential businesses have been closed. Consequently, frequencies of both labor-related injuries, violence, and traffic incidents were likely lower than during normal conditions. It is assumed that admission rates of trauma patients have been influenced by patients avoiding trauma submissions.

The data collected in this study revealed that “*Group Pan”* had significantly lower ISS, compared to the reference group from the year 2019 “*Group Ref*.” It is tempting to hypothesize that increased awareness and alertness of the general population might have affected behaviors towards high-risk sports or driving habits. However, the number of severely injured patients was comparable between the groups, indicating the necessity of maintaining the same quality of care despite the decrease of ISS.

Even with a reduced injury severity, an increased duration from admission to CT scan was observed. These changes are difficult to explain; however, they might be a result of increased alertness of medical personnel, associated with increased hygiene recommendations and personal protection measures. The increased caution of self-exposure towards potentially infected people during daily living might also have affected medical personnel during their routine clinical treatment.

Based on these changes and the developments during the SARS CoV-2 crisis, the following points should be discussed regarding potential adjustments to existing trauma bay guidelines (Table 3):

**Table 3:** Suggested adjustments of trauma bay protocols during endemic COVID-19

Medical personnel	Appropriate Team Training to use PPE
	Consider every patient as infected
	Minimize direct contact and define “hands-on” and “hands-off” teams
	Define strategies in treatment of two and more patients simultaneously
Diagnostics	Cohort infected patients if possible
	Avoid transportation through hospital
	Use portable imaging
	Reduce or avoid repeated evaluations
	No face to face consultations
General management	Avoid second hit phenomenon in infected
	• Minimize interventions “damage control”
	• Adequate prevention of thrombosis
	• Avoid secondary infection
	• Lung-protective ventilation

*PPE* personal protective equipment

## Medical personnel in trauma bay

**Fig. 1:**
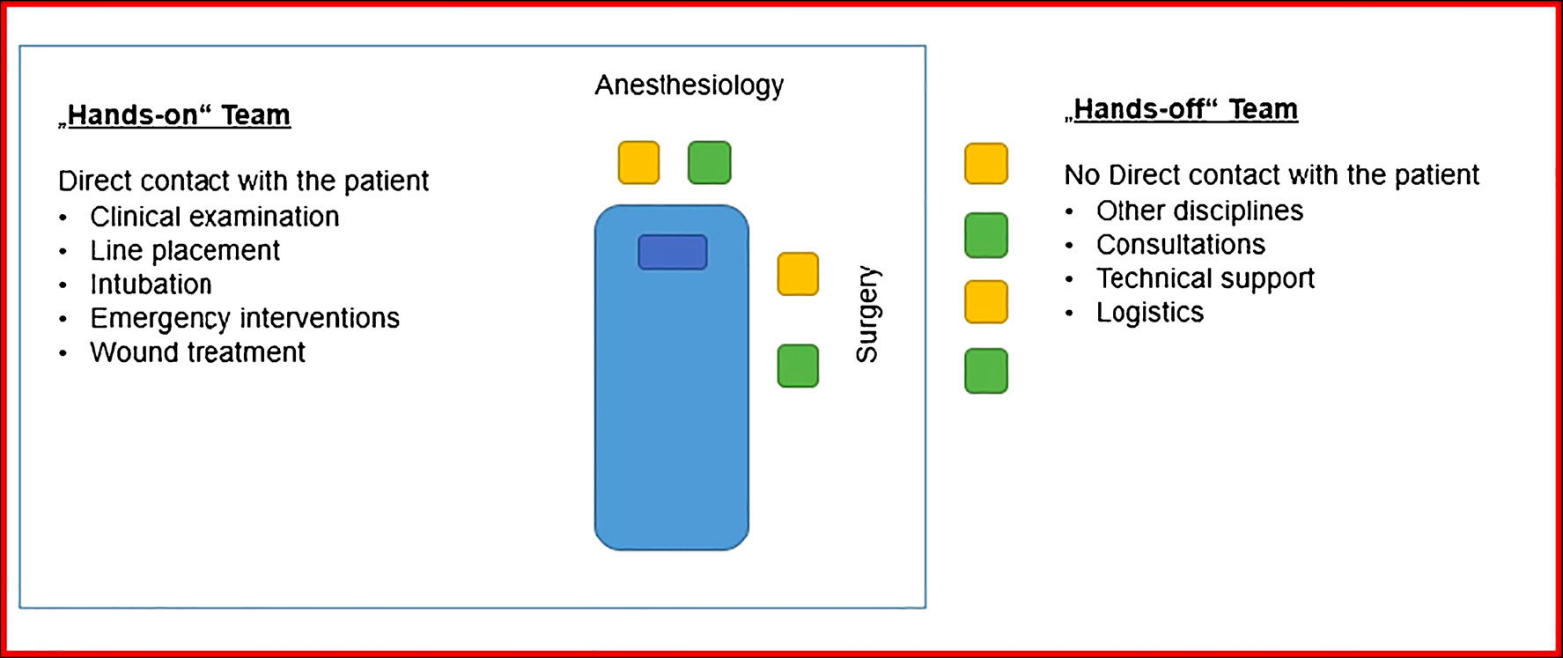
The whole treatment team could be grouped in two teams: hands-on and hands-off teams. The hands-on team has a direct contact with the patient and performs clinical examination and emergency interventions. The hands-off team avoids the direct contact with the patient and contaminated materials

Recent guidelines (Level 3 Guidelines on the treatment on patients with severe/multiple injuries by European Society of Trauma and Emergency Surgery (ESTES)) indicate that the basic trauma team must consist of at least three physicians (2 surgeons and 1 anesthesiologist) [6, 11]. Local conditions of the trauma system in each region usually define which specialties are primarily present in the emergency department. Other disciplines, such as neurosurgeons or/and radiologist, and nurses/technicians from each department are frequently present in emergency department during the initial assessment of the patient in the trauma bay. In the time of pandemics, such as COVID-19, where there is a high infection rate, all patients need to be initially treated as infected until proven otherwise [12]. The number of medical personnel with direct contact to the patient needs to be decreased during such pandemics in order to increase patient and medical personnel safety [13]. Moreover, the trauma team needs appropriate training in the safe use of personal protective equipment (PPE) [14]. In order to avoid cross-contamination, the exact roles in trauma bay need to be adjusted for pandemics such as COVID-19 [15]. Physicians with direct contact to the patient (e.g., anesthesiologists and surgeons) may be defined as the “hands-on” team, whom perform the line placement, blood collection, and clinical examination or emergency interventions (Fig. 1). Depending on the injury pattern or severity, the initial team can be supplemented (hands-off team) with additional colleagues (e.g. neurosurgeons, vascular surgeons, thoracic surgeons etc.) (Fig. 1). The function and the role of a “trauma leader” stays unaffected according to each system and organization. Especially, if the treatment of more than one severely injured patients simultaneously requires a clear definition of teams and consideration of hygiene standards. Moreover, the above-mentioned strategies lead to a rationalized use of personal protective equipment, such as gloves, masks, and glasses.

## Diagnostics

Severely injured patients are frequently subjected to different diagnostic modalities to identify life-threatening injuries and patterns. Chest and pelvis X-rays, sonography, whole-body computed tomography (WBCT), and angiography are commonly used diagnostic procedures in trauma bay. The evaluation of the trauma patient should not be delayed, and appropriate precautions should be taken. When possible, diagnostic procedures are performed at sites with less traffic of outpatients to avoid secondary exposure of patients and medical staff. When possible, COVID-19 patients should be placed in a separate location from non-COVID-19 patients; however, optimal injury care should be a priority [16]. Transportation of trauma patients through the hospital should be avoided. Therefore, whenever possible, the use of portable imaging, such as portable x-rays and sonography, is recommended. Every patient is assumed to be infected; therefore, adequate decontamination of imaging equipment and any surface is necessary. When possible, repeated examinations should be reduced or entirely avoided with initial adequate diagnostics. The number of patient transfers to imaging facilities can be reduced by performing all imaging directly upon admission. In order to increase capacity, non-urgent imaging can be delayed or postponed. Discussions of imaging referrals during face-to-face consultations have a potential risk of contamination, and it is recommended that this is done over the phone.

## General management

Recent publications indicate that a relevant number of SARS-CoV-2-infected patients may develop severe inflammatory complications with diffuse pulmonary inflammation and alveolar damage [17, 18]. Some reports even document the presence of a cytokine storm in the infected patients leading to altered cellular and humoral immune responses [19–21], similar to post-traumatic acute lung injuries (ALIs) induced by both direct and non-direct pulmonary insults [22, 23]. Besides the assessment of the overall conditions of trauma patients (age, injury severity, pattern, physiological response), the presence of bilateral opacities in chest imaging may influence the decision-making in trauma bay, as well. The surgical management should be based on minimizing the “secondary hit” phenomena to the immune response [24, 25]. In addition, lung-protective ventilation protocols may prevent barotrauma. Mechanical ventilation can exacerbate lung damage by causing a secondary induced lung injury when used improperly [26]. The prevention of secondary infections is also stressed in order to prevent additional infectious insults and inflammatory exaggerations. Moreover, recent publications indicate high rates of thromboembolic complications in COVID-19-infected patients [27]. Therefore, adequate anticoagulation is recommended according to patient conditions.

## Conclusion

The worldwide COVID-19 pandemic has had an immense impact on the day life and clinical practices. The number of patients admitted to the trauma bay has decreased; however, the number of severely injured trauma victims was unchanged. In order to maintain the quality standards, adjustment to the current trauma guidelines for pandemic situations is needed. Patient and personnel safety should be the number one priority in such situations. Moreover, the above-mentioned strategies could also lead to improved and sustainable use of personal protective equipment.

## Data Availability

All data and material are available upon request from the corresponding author. The datasets generated during and/or analyzed during the current study are available in the figshare repository 10.6084/m9.figshare.12436814
